# Pathogen-Mediated Alterations of Insect Chemical Communication: From Pheromones to Behavior

**DOI:** 10.3390/pathogens12111350

**Published:** 2023-11-14

**Authors:** Andrea Moyano, Anna Cleta Croce, Francesca Scolari

**Affiliations:** 1 Institute of Molecular Genetics, Italian National Research Council (CNR), Via Abbiategrasso 207, I-27100 Pavia, Italy; andrea.moyano@igm.cnr.it (A.M.); annacleta.croce@igm.cnr.it (A.C.C.); 2 Department of Biology and Biotechnology, University of Pavia, Via Ferrata 9, I-27100 Pavia, Italy

**Keywords:** bacteria, viruses, fungi, eukaryotes, beneficial insect species, insect control

## Abstract

Pathogens can influence the physiology and behavior of both animal and plant hosts in a manner that promotes their own transmission and dispersal. Recent research focusing on insects has revealed that these manipulations can extend to the production of pheromones, which are pivotal in chemical communication. This review provides an overview of the current state of research and available data concerning the impacts of bacterial, viral, fungal, and eukaryotic pathogens on chemical communication across different insect orders. While our understanding of the influence of pathogenic bacteria on host chemical profiles is still limited, viral infections have been shown to induce behavioral changes in the host, such as altered pheromone production, olfaction, and locomotion. Entomopathogenic fungi affect host chemical communication by manipulating cuticular hydrocarbons and pheromone production, while various eukaryotic parasites have been observed to influence insect behavior by affecting the production of pheromones and other chemical cues. The effects induced by these infections are explored in the context of the evolutionary advantages they confer to the pathogen. The molecular mechanisms governing the observed pathogen-mediated behavioral changes, as well as the dynamic and mutually influential relationships between the pathogen and its host, are still poorly understood. A deeper comprehension of these mechanisms will prove invaluable in identifying novel targets in the perspective of practical applications aimed at controlling detrimental insect species.

## 1. Introduction

Pathogens can manipulate host physiology and behavior to their benefit across the tree of life, with effects reported from plants to animals, including both vertebrates and invertebrates [1,2,3,4,5,6,7,8,9,10,11]. The effects of this manipulation are extremely variegated. In plants, for instance, bacterial pathogens can hijack the host physiology, affecting its metabolites and immune proteins to promote their own dissemination through increased attraction of insect vectors [9,10,12], or they can alter hormonal signaling and susceptibility, augmenting pathogen growth and its ability to evade host defensive responses [11]. In land snails, parasitic flatworms can concentrate in the host eye stalks and pulsate to attract snail bird predators, which are the primary flatworm hosts [7]. Similarly, the manipulations induced in rats and mice by *Toxoplasma gondii* (Eucoccidiorida: Sarcocystidae), an obligate intracellular protozoan able to reduce their fear of cats and increase the chances of being predated, favors the transfer of the parasite to the cat, its primary host [13,14,15,16]. These effects are achieved through the modification of vasopressin and dopamine metabolism and the consequent epigenetic brain reprogramming. Hairworms (Nematomorpha) can alter crickets’ behavior, making them jump into water, thus enabling the hairworms to reach their reproductive habitat [5]. The fungus *Ophiocordyceps unilateralis* (Hypocreales: Ophiocordycipitaceae), a parasite of *Camponotus leonardi* Emery, 1889 (Hymenoptera: Formicidae) ants, is known to induce its hosts to reach an elevated position (i.e., summiting behavior) and bite into vegetation before death [17]. This extended behavioral phenotype [18,19] allows the fungus fruiting body to grow, ensuring widespread spore dispersal [20]. Ants of the species *Formica fusca* Linnaeus, 1758 (Hymenoptera: Formicidae) can be infected by another parasite, the lancet liver fluke *Dicrocoelium dendriticum* (Rudolphi, 1819) (Plagiorchiida: Dicrocoeliidae), a trematode that can take over the host’s navigational abilities until the ant is predated by grazing animals [21,22]. Analogous suicidal behaviors are induced by infections of *Mermis* nematodes in parasitized ants, which, after infection, search for water, jump in, and drown [23]. Similar behavioral phenotypes have been recently observed in several other summit disease systems, including those mediated by fungal and baculovirus infections (see [24] for a review). The mechanisms underlying such peculiar phenotypes are poorly understood and recent multi-omic approaches are beginning to reveal a dysregulation of neuronal signaling and the levels of neurotransmitters [25]. Numerous fungal molecules have been proposed to interact with ant molecular pathways, resulting in behavioral changes such as modified locomotion, feeding, light-sensing, circadian rhythms, and muscular hyperactivity [26,27,28,29,30,31,32]. The use of machine learning to predict host–parasite protein–protein interaction (PPIs) is an emerging approach aimed at generating fresh insights into the mechanisms behind these behavioral alterations, as recently shown by Will and colleagues [33]. In this study, PPIs involving fungal S8 proteases, oxidation-reduction processes, gene regulation, GPCRs, and cuticular proteins have surfaced as promising candidates for functional validation, providing new insights into co-evolved adaptations that underlie these modified behaviors.

Pathogens have also demonstrated the ability to manipulate the central mechanisms in the brain of vertebrates, thereby altering social interactions, including aggressive, reproductive, and parental behaviors, to promote their own transmission [34]. Intriguingly, recent research has revealed that infection with the bacterial pathogen *Mycoplasma gallisepticum* Edward and Kanarek, 1960 (Mycoplasmatales: Mycoplasmataceae) can lead to an increase in social preferences among juvenile house finches *Haemorhous mexicanus* (P.L. Statius Muller, 1776) (Passeriformes: Fringillidae) [35]. Specifically, infected finches spent more time associating with healthy flocks compared with uninfected individuals, with significant implications for *Mycoplasma* transmission. Additionally, male guppies (*Poecilia reticulata* Peters, 1859 (Cyprinodontiformes: Poeciliidae)) hosting high loads of the flatworm ectoparasite *Gyrodactylus turnbulli* Harris, 1986 (Gyrodactylidea: Gyrodactylidae) exhibited increased sociality, possibly to enhance their mating opportunities and consequently transfer the parasite onto other hosts [36].

Pathogen-induced alterations in social behavior are not limited to vertebrates, they also extend to invertebrates. An example of this phenomenon can be observed in crustacean hosts (genus *Artemia* (Anostraca: Artemiidae)), which, when infected with two microsporidian parasites, show increased swarming and surfacing propensity, resulting in augmented spore transmission to new hosts [37]. In a recent study, adult *Caenorhabditis elegans* (Maupas, 1900) (Rhabditida: Rhabditidae) hermaphrodites exposed to the bacterial pathogen *Pseudomonas aeruginosa* (Schroeter 1872) (Pseudomonadales: Pseudomonadaceae) displayed a modulated sensory response to pheromones [38]. Considering that ascaroside pheromones repel hermaphrodites and attract males, this avoidance was shown to facilitate individual dispersal, promoting mating with a potential adaptive role for the host, as outcrossing may be advantageous over selfing in certain environmental conditions. This study shed new light on the roles played by pathogens in inducing social behavior plasticity, which can increase genetic diversity and favor host adaptation. The documented instances of increased sociality are thus not only advantageous for the pathogen but they might also ultimately enhance host fitness. This underscores the intricacy of such infection-related behavioral changes, which can be driven by the host or the pathogen, or can be a combination of both types of manipulations [39].

Pathogen-induced behavioral changes represent an interesting research area in entomology, with ecological and practical implications for the preservation of beneficial species and the management of agricultural pests and disease vectors. For instance, fungal infections can impact host thermal tolerance, as shown in the system involving the fungal pathogen *Beauveria bassiana* (Bals.-Criv.) Vuill., 1912 (Hypocreales: Cordycipitaceae), the herbivorous insect *Acyrthosiphon pisum* (Harris, 1776) (Hemiptera: Aphididae), and its predator beetle *Hippodamia convergens* (Coleoptera: Coccinellidae) [40]. In this case, fungal infection reduced the heat tolerance of both the pea aphid and the beetle. Conversely, the effects on cold tolerance were species-specific, with reduced cold tolerance found only in *H. convergens*. This example illustrates how pathogens can play a role in shaping predator–prey interactions and food webs, carrying implications for insect control. Furthermore, parasite infection may increase insect activity, potentially leading to higher parasite dispersion and, consequently, higher transmission rates. This is exemplified by the case of the nymphs of the triatomine bug *Rhodnius prolixus* (Hemiptera: Reduviidae), which exhibit increased locomotor activity when infected with *Trypanosoma rangeli* Tejera, 1920 (Trypanosomatida: Trypanosomatidae) [41]. Along the same lines, the mosquito vector *Aedes aegypti* (Linnaeus, 1762) (Diptera: Culicidae), when infected with a virulent strain of *Wolbachia pipientis* Hertig, 1936 (Rickettsiales: Anaplasmataceae) able to shorten insect lifespan, displays increased activity and metabolic rates [42]. In the same mosquito species, infection with the dengue virus DENV-1 not only affects locomotion but also odor-mediated behavior. The mosquito peripheral olfactory system is altered by this arbovirus infection, with enhanced antennal neural responsiveness resulting in improved mosquito host-seeking capacity and, consequently, increased virus transmission risks [43].

Chemical communication is fundamental to fulfil insect needs, such as locating food, mates, and oviposition sites, as well as avoiding predators and detrimental microbes. In this context, pheromones play a well-established role. Pheromones are generally defined as molecules capable of mediating communication between conspecifics [44], and they serve a wide spectrum of functions, including sexual interaction, aggregation, alarm signaling, and trail and host marking [45,46]. Pheromones are typically mixtures of different compounds and their composition is strongly influenced by various factors, including diet and endosymbionts (for a comprehensive review, see [47] and references therein). The important role of microbiota on the physiology and ecology of insect species is a widely described phenomenon. It comprises nutritional symbiosis, provision of bioactive compounds, protection against toxins and parasitoid wasps, enhanced resistance towards fungal and bacterial pathogens, pesticide detoxification, and effects on prey–predator interactions [48,49,50,51,52,53,54,55,56,57,58,59,60]. Interestingly, symbionts can also influence insect chemical communication. For example, endosymbionts can produce volatiles used as sex pheromones [61,62,63,64] or aggregation pheromones [65,66], other can convert pheromones into repellents [67], or can participate in the metabolism of cuticular hydrocarbons [68].

Conversely, there is still limited information regarding the influence of insect parasites and pathogens on the alteration of host pheromone production and, thus, their communication capacity [48]. Expanding our understanding in this field is crucial for gaining insights into the impact of pathogen infections on the biology of insect hosts.

In this review, we aim to provide an up-to-date overview of the literature that explores the impact of pathogens on insect pheromone production and the related sexual behavior. These findings are described in the context of the adaptive role of these manipulations, which are generally known to alter host behavior in a way that enhances the likelihood of pathogen transmitting [69].

This topic has so far been investigated in insect hosts belonging to the Diptera, Lepidoptera, Hemiptera, Hymenoptera, Orthoptera, Blattodea, and Coleoptera orders, as summarized in Table 1.

Achieving a wider understanding of the pheromone-mediated behavioral changes triggered by pathogen infections also offers substantial potential for the development and improvement of tools for insect pest and vector control within integrated pest and vector management (IPM and IVM, respectively) approaches. Given that environmentally friendly insect control strategies often rely on communication disruption methods (see [106] for a review), exploring the molecular underpinnings of pathogen-mediated behavioral changes is a promising research field.

The following sections of this review will illustrate the existing knowledge concerning the impact of pathogens belonging to bacteria, viruses, fungi, and eukaryote taxa on the chemical communication of their insect hosts. The described effects are examined within the context of the evolutionary advantages they provide to the pathogen, as well as in relation to the life history traits that insect hosts can compromise in their efforts to counteract pathogen infection.

## 2. Bacteria

Although *Drosophila* is considered a powerful model to study the host immune, hormonal, and metabolic responses to pathogenic bacteria [107,108,109,110,111,112,113,114,115,116], the specific effects these infections cause on pheromone production and, consequently, on chemical communication are still largely unexplored.

So far, only one study showed that the infection of pathogenic bacteria can induce a pheromone change in *D. melanogaster* Meigen, 1830 (Diptera: Drosophilidae) [70]. Indeed, gas chromatography mass spectrometry (GC-MS) analyses of the volatile and non-volatile chemicals emitted by *Drosophila* infected by pathogenic and non-pathogenic bacteria resulted in sharply different results. Infection with the non-pathogenic bacteria *Lactobacillus plantarum* (Orla-Jense 1919) (Lactobacillales: Lactobacillaceae) and *Acetobacter pomorum* Sokolle et al., 1998 (Rhodospirillales: Acetobacteraceae) and the facultative endosymbiont *Wolbachia* did not result in differences in the odor profile. Conversely, infection with the natural bacterial pathogens *Pseudomonas entomophila* Mulet et al., 2012 (Pseudomonadales: Pseudomonadaceae), *Serratia marcescens* Bizio, 1823 (Enterobacterales: Yersiniaceae), and *Erwinia carotovora carotovora* (*Pectobacterium carotovorum* (Jones, 1901) Waldee, 1945 (Approved Lists, 1980) emend. Portier et al., 2019) (Enterobacterales: Pectobacteriaceae)) strongly affected the chemical profile of both male and female adult flies. These pathogenic manipulations affected olfactory cues related to both attraction and aggregation. Infected flies were found to release increased levels of chemicals, including the aggregation pheromones methyl laurate, methyl myristate, and methyl palmitate. These volatiles were demonstrated to attract healthy flies, thereby facilitating the further spread of the pathogen once the healthy flies became infected.

In the same study, authors also tested *P. entomophila* infection in other dipteran species, namely eight other Drosophilids, the blue bottle fly, *Calliphora vomitoria* (Linnaeus, 1758) (Diptera: Calliphoridae), and the two mosquitoes *Aedes aegypti* and *Culex pipiens* Linnaeus, 1758 (Diptera: Culicidae). The infections proved lethal for all the tested species. However, intriguingly, a significant increase in the emissions of potential fatty-acid pheromones was observed in seven *Drosophila* species and *Ae. aegypti*. This discovery paves the way for further exploration into the potential conservation of pheromone manipulations induced by *P. entomophila* across distantly related insect species.

To the best of our knowledge, no additional effects of pathogenic bacteria on insect chemical communication and, consequently, their behavior have been reported thus far, rendering this research field largely unexplored.

## 3. Viruses

Viral replication has been observed to influence the reproductive physiology and behavior of infected insects, indicating the existence of a co-evolutionary relationship between the virus and the host aimed at facilitating transmission. This is the case of the rod-shaped enveloped virus *Helicoverpa zea* nudivirus 2 (Hz-2V) (Lefavirales: Nudiviridae), found to be able to persistently infect a colony of *Helicoverpa zea* (Boddie, 1850) (Lepidoptera: Noctuidae), the corn earworm moth [8,117,118]. This virus can be horizontally transmitted during mating [118]. In infected females, the virus primarily replicates in the oviducts, where the viral particles accumulate with a matrix, forming a plug of virus-filled vesicles located on the tip of the vulva [119] that contributes to further infection upon mating with healthy males [120]. Interestingly, viral replication in reproductive tissues results in insect malformations and sterility (agonadal condition) [119,121,122], with effects on insect behavior. Indeed, in flight tunnel assays, agonadal females exhibit calling, producing five to seven times more pheromones than controls, resulting in attraction of more males than those attracted by uninfected females [8]. This increased attractiveness of infected females may facilitate viral transmission across insect populations. Moreover, infected agonadal males lack accessory glands and cannot produce the pheromonostatic peptide (PSP) that, when transferred to females upon mating, induces a strong decrease in female pheromone titer. These effects thus negatively affect calling behavior [72] and favor viral transmission [71].

Viral infection can induce alterations in host perception by modulating the expression of olfactory genes. Notably, infection by baculoviruses, which are double-stranded DNA entomopathogenic viruses, has recently been shown to affect the olfaction of the host. This effect is likely related to the enhanced locomotion activity shown by baculovirus-infected larvae of *Bombyx mori* Linnaeus, 1758 (Lepidoptera: Bombycidae), suggested to promote viral dispersion [123]. The multiple nucleopolyhedrovirus of *Spodoptera exigua* (SeMNPV) can induce changes in the expression of some odorant receptors (ORs) during the larval stage [73]. The functional characterization of *SexiOR35*, which was strongly upregulated upon SeMNPV infection, revealed a link between the change in transcription and infection-related shifts in larval behavioral responses to linalool and estragole, two of its main ligands. Whether the observed changes resulted from a specific effect of infection or a related side effect remains to be determined.

The baculovirus *Autographa californica* multiple nucleopolyhedrovirus (AcMNPV) is known to cause systemic infections in over 35 lepidopteran species [124]. Among the insect species most vulnerable to AcMNPV infection, we can find the serious agricultural pests *Spodoptera frugiperda* (Smith, 1797) (Lepidoptera: Noctuidae), the fall armyworm, and *Trichoplusia ni* (Hubner, 1803) (Lepidoptera: Noctuidae), the cabbage looper. The infection of this baculovirus via horizontal transmission derived from virus consumption has fatal effects, leading to the liquefaction of the insect larval host [125]. In addition, the infection of the midgut epithelial cells results in secondary infection in the open circulatory system, favoring invasion of other tissues and hampering larval growth or molting [74]. The genetic bases of AcMNPV infection are still poorly characterized but are extremely important to be unraveled to exploit the biocidal activity of AcMNPV on its hosts. In this view, interesting perspectives have been provided by a recent study that generated transcriptomes from the hemolymph of AcMNPV-infected *S. frugiperda* and *T. ni* fourth instar larvae [74]. In infected hosts, chitin metabolism, tracheal development, and immunity genes were found to be transcriptionally suppressed, and induction of oxidative stress indicated disease progression in the insect hosts [74]. Interestingly, the genes *Desaturase1* (*Desat1)*, known to have effects on *Drosophila* pheromone production and perception [126,127] and *bond*, which has roles in conspecific signaling [128], are co-induced in infected hosts [74]. In addition, functions related to fatty acid biosynthesis and pheromone metabolisms are observed to be enriched in response to AcMNPV infection, similar to the trend detected in other insect species, such as *Drosophila* [75] and *Aedes aegypti* [76], as explained below. The finding that pheromone signaling pathways appear to be generally inducted during viral infection in different insect models suggests that the pathogen may exploit them to favor host aggregation, thus facilitating viral spread and, consequently, disease progression among individuals. On this basis, Pantha and colleagues suggest that the pleiotropic gene Desat1 may be a good candidate for future studies as it may be co-opted for behavioral traits evolved in the context of the co-evolution between lepidopteran hosts and baculoviruses [74].

Additional studies used the *Drosophila* model to investigate response to viral infection. For example, Sabatier and colleagues developed a model using the *Drosophila* C virus (DCV) [75]. *Drosophila* is a natural host for this pathogen, which is a non-enveloped small single-stranded (+) RNA virus transmitted horizontally [129,130,131] and that was found to induce one peptide, namely pherokine-2 (Phk-2), in the hemolymph of infected flies [75]. In the same study, pherokine-3 (Phk-3) was found to be induced by bacterial challenge. On this basis, and since these pherokines correspond with the products of a gene related to sequences specifically expressed in the antennal olfactory region, a potential role of the sensory system in host-defense in *Drosophila* has been suggested [75,132], as occurring in social insect species [133,134]. However, the overexpression of Phk-2 in transgenic flies was not found to increase protection against infection with DCV [75].

In this context, an additional recent study focused on analyzing the effects of a human pathogenic arbovirus on the olfaction of *D. melanogaster.* Infection with the Rift Valley fever virus (RVFV) resulted in decreased antennal responses to food-related odorants and reduced locomotor activity, potentially due to a direct effect of the virus on the host nervous system [77]. This study provides a novel perspective in the field, indicating *Drosophila* as a useful model to investigate arbovirus–vector interactions.

Among the human pathogenic arboviruses of major public health importance, the dengue virus DENV-2 has been reported to affect the expression of chemosensory-related genes that regulate blood feeding in its vector, the mosquito *Ae. aegypti* [76]. Two odorant-binding proteins (OBPs) identified as responsive to DENV-2 infection seem to play a role in the probing process, as their silencing negatively affects blood acquisition. This viral induction of OBPs could theoretically increase viral transmission efficiency.

The *Rhopalosiphum padi* virus (RhPV) (Picornavirales: Dicistroviridae) can infect aphids (Hemiptera: Aphididae) and affect their biology, including reduction of longevity and fecundity [135]. In the aphid *Rhopalosiphum padi* (Linnaeus, 1758) (Hemiptera: Aphididae), uninfected individuals were observed to be attracted to the odor emitted by other uninfected aphids, leading to aggregation on the host plant. In contrast, infected individuals did not exhibit this attraction and failed to respond to cues, indicating host suitability. Interestingly, infected aphids were more sensitive to the alarm pheromone [78]. Additionally, the ladybird *Coccinella septempunctata* Linnaeus, 1758 (Coleoptera: Coccinellidae) exhibited a higher predation rate on infected aphids compared with uninfected individuals. The aphid parasitoid *Aphidius ervi* Haliday (Hymenoptera: Braconidae) demonstrated a preference for attacking infected insects. These observations underscore the potential interference of pathogens with insect chemical communication. The question of whether the reported effects are advantageous for the aphid or the virus remains an open inquiry.

## 4. Fungi

In comparison to other pathogens, the infection of fungal pathogens in insects has received more extensive attention. The reported effects on the host primarily involve altered pheromone production, modification of volatile organic compounds (VOCs), and changes in cuticular hydrocarbon (CH) profiles.

Adults of *Triatoma infestans* (Hemiptera: Reduviidae) can secrete a mixture of VOCs with alarm function and potential sexual and defensive roles [79,136,137,138,139]. Lobo and colleagues observed that *T. infestans* infected with the entomopathogenic fungus *B. bassiana* showed differences in the VOCs’ profiles [80]. In particular, the amount of propionic acid, a major component of the alarm pheromone in this species, was up to three-fold higher 1–4 days post-infection with respect to its level in uninfected individuals. In addition, the two genes *Ti-brnq* and *Ti-bkdc*, involved in the volatile short-chain fatty acid synthesis, increased their expression in individuals in the early stages of fungal infection. These data contribute to shedding light on the chemical ecology of triatomine bugs, a research field of particular importance, especially from an applied perspective in vector control, given the major role of *T. infestans* as a vector of the causative agent of Chagas disease in the Southern region of South America.

The obligate specialist pathogen of aphids, *Pandora neoaphidis* (Entomophthorales: Entomophthoraceae), has been suggested to face higher selection pressure to enhance its transmission and subsequent survival compared with *B. bassiana*, a generalist pathogen capable of surviving as a saprotroph in the soil while also acting as a pathogen with a broad host range [89]. Indeed, *Acyrthosiphon pisum* (Harris, 1776) (Hemiptera: Aphididae) aphids infected with *B. bassiana* exhibited a reduced production of alarm pheromones compared with uninfected hosts. In contrast, aphids infected with *P. neoaphidis* released more alarm pheromones than the uninfected controls, resulting in opposing effects on the movement ability of uninfected individuals. These differences may lead to infected aphids falling to the soil (due to decreased movements) or having increased chances of contact with fungal conidia (due to increased movements), both of which contribute to favoring pathogen dissemination and host infection in the context of their respective generalist or obligate life history strategies [89].

Alterations in the pheromone profile of honeybee workers and queens have been noted when infected with the microsporidian fungal gut pathogen *Nosema ceranae* Fries, Feng, da Silva, Slemenda and Pieniazek, 1996 (Apansporoblastina: Nosematidae) [82,84,85]. Initially, the effect of this parasite on pheromone signaling, particularly in the production of ethyl oleate (EO), the only primer pheromone involved in foraging so far identified in honeybee workers, was analyzed in this caste. The authors found that *Nosema* spp. could significantly alter the production of EO, with a positive correlation between the levels of *Nosema* infection and EO synthesis [82]. These results suggest that *Nosema* infection may affect colony homeostasis due to the involvement of this compound in the regulation of labor division among workers. Moreover, when *N. ceranae* challenged bees in the field, higher EO levels and increased flight activity were observed, along with high mortality compared with uninfected individuals [83]. Higher flight activity by *Nosema*-infected bees might help reduce disease transmission rates within the colony. However, as the chemical mechanisms regulating behavioral maturation, particularly the balance between nursing and foraging roles, become disrupted, the colony displayed a more fragile homeostasis, making it more susceptible to other environmental disturbances [83].

When queens were investigated for the effects of *N. ceranae*, Alaux and colleagues found that infection resulted in increased vitellogenin titer, antioxidant capacity, and mandibular pheromones, potentially impacting their health [84]. The profile of the queen mandibular pheromone (QMP) could indeed be indicative of infection-related increased frequencies of its supersedure. In particular, 9-oxodec-2-enoic acid (9-ODA) and 9-hydroxy-2-enoic acid (9-HDA) levels were found to be significantly higher, while the antifungal compound methyl p-hydroxybenzoate (HOB), a potential signal prompting initiation of queen rearing, decreased in infected queen heads [84].

Mayack and colleagues recently used a high-resolution accurate mass gas chromatography–quadrupole time-of-flight mass spectrometry approach to trace the exposome profile of hives where *N. ceranae* was found to be present [86]. The significant correlation detected between the presence of *N. ceranae* infection and one component of the alarm pheromone, namely (*Z*)-11-eicosen-1-1ol, suggested that the increase in the synthesis of this compound may represent a recognition mechanism for the colony to identify, care for, quarantine, or kill the infected individuals. Research in this field is of particular interest, since *N. ceranae* infection contributes to the global decline in the health of honeybees. A deeper understanding of the mechanisms through which a colony fights against the disruption of its social harmony using alarm pheromones is also essential from the applied point of view.

The important threat to food security posed by locusts is based on their capacity to aggregate into migratory swarms thanks to a complex interaction of visual, tactile, and chemical cues. *Paranosema locustae* (Apansporoblastina: Nosematidae) infection can inhibit the aggregation behavior of solitary *Locusta migratoria manilensis* (Meyen, 1835) (Orthoptera: Acrididae) and induce the return to solitary lifestyles by gregarious individuals [87,88]. Shi and colleagues found that this behavioral change is related to a reduction in the bacterial population contributing to the production of the aggregation pheromone in the locust hindgut because of the acidification and increased production of reactive oxygen species induced by *P. locustae* [88]. These findings are thus of particular interest in developing improved strategies for locust control by exploiting the interactions between the parasite and the gut microbiota.

Cuticular hydrocarbons are key components of the thin film of wax covering the body surface of many insect species [140]. In addition to their waterproofing function, CHs are involved in chemical communication and have been widely explored in many taxa [47,141,142,143,144]. Cuticular hydrocarbons are mainly synthesized in the oenocytes, specialized secretory cells associated with the epidermis or the fat body and rich in endoplasmic reticulum and mitochondria [145]. The properties of these cells align with their common features across insect species, consisting of the involvement in the regulation of lipid metabolism [146,147,148,149]. The biochemical steps of CH biosynthesis have been widely described and are based on the highly conserved pathway of fatty acid production [140,150,151,152,153]. Briefly, fatty acid synthase generates fatty acyl-CoA through malonyl-CoA. Then, the fatty acyl-CoA is extended by elongases, and double bonds are introduced by desaturases, leading to the synthesis of unsaturated hydrocarbons, most commonly alkadienes and *n*-alkenes [153]. Reductases then convert the acyl-CoA to aldehydes, which serve as substrates for a single carbon chain-shortening conversion to hydrocarbons, a reaction catalyzed by P450 enzymes from the CYP4G subfamily [154,155,156,157,158]. After being synthesized, the CHs are transported through the hemolymph by lipophorin and are subsequently carried to the epicuticular surface through specialized pore canals crossing the cuticular layers [159,160]. The mechanisms underlying the transport of CH precursors amino acids and propionate into the oenocytes have not been completely understood thus far [159], and how their engagement in producing CHs might adapt as a response to pathogen infections is still to be explored.

Some fungi have the capability to penetrate the CH mechanical barrier [161,162], leading to alterations in the CH content of the infected insect species. In order to penetrate the insect cuticle, entomopathogenic fungi exploit a complex mixture of enzymes [163,164,165,166], including cytochrome P450 monooxygenases, which mediate the degradation of CHs. This effect has been documented in the context of *T. infestans* infection with *B. bassiana* and *Metarhizium anisopliae* (Metschn.) Sorokin, 1883 (Hypocreales: Clavicipitaceae). In these cases, the fungi were shown to convert CHs into different lipid products, serving as a source for energy production and the biosynthesis of cell components [167,168]. Moreover, the larvae of the common cockchafer *Melolontha melolontha* (Linnaeus, 1758) (Coleoptera: Scarabaeidae) and the European corn borer *Ostrinia nubilalis* (Hubner, 1796) (Lepidoptera: Crambidae) showed altered CH profiles following treatment with *B. bassiana* or *B. brongniartii* (Sacc.) Petch, 1926 (Hypocreales: Cordycipitaceae) [81]. This is particularly interesting, since *M. melolontha* is not a natural host for *B. brongniartii*, showing that exposure to non-infective spores may also impact CHs and, as a potential consequence, host chemical communication.

While certain entomopathogenic fungi have been demonstrated to influence the overall behavior and neurological patterns of infected individuals to their advantage (e.g., increased conidial transmission) [20], there are very few studies to date examining the effects of entomopathogenic fungi on altering insect chemical communication and, consequently, sexual behavior [169]. This is the case of the cockroach *Blatta orientalis* Linnaeus, 1758 (Blattodea: Blattidae), which produces more hydrocarbons and other surface compounds after exposure to the entomopathogenic fungus *Conidiobolus coronatus* (Entomophthorales: Ancylistaceae) [90]. In ants of the species *Lasius neglectus* Van Loon, Boomsma, and Andrsfalvy, 1990 (Hymenoptera: Formicidae), when the pupae are infected with *M. brunneum* Petch, 1935 (Hypocreales: Clavicipitaceae), the CH profiles change and the altered chemical cues they emit during the non-transmissible incubation time of the pathogen trigger a destructive disinfection behavior [91]. Ants use their antimicrobial poison as well as cocoon removal and biting to avoid the replication of the pathogen in the pupae, thus interrupting the pathogen lifecycle. In the case of *Nosema apis* E. Zander, 1909 (Apansporoblastina: Nosematidae) and *N. ceranae* infection in honeybees, Murray and coworkers found alterations in the *n*-alkane profile but did not detect altered behaviors in the nestmates [170]. Research in this field is still at an early stage and indicates the presence of adaptive roles of CH changes upon entomopathogenic fungi infection either for the host, which can detect the infection and remove it efficiently, or for the pathogen, which can degrade the CH layer.

In some cases, the advantage for the pathogen (e.g., ensuring its dispersal) is particularly evident. An example is the pathogenic fungus *Entomophthora muscae* (Entomophthorales: Entomophthoraceae), which can produce a mixture of volatile sesquiterpenes altering the natural profile of the CHs of cadavers of female house flies, *Musca domestica* (Diptera: Muscidae) [92]. Uninfected males are then attracted by the fungal-produced compounds and mate with the dead females, thus increasing the chances of being infected and contributing to pathogen transmission.

The advantage for the pathogen may also occur at the metabolic level, such as in the synthesis of epicuticular hydrocarbons in the insect host and their assimilation by entomopathogenic fungi. Further exploration of this interaction could provide valuable insights into a potentially significant case of pathogen–host co-evolution. Additionally, it may aid in identifying novel molecular targets in the biochemistry of CH degradation, offering key biotechnological applications for controlling harmful insect species [168].

## 5. Eukaryotes

### 5.1. Phylum Apicomplexa

The malaria parasite *Plasmodium falciparum* (Haemosporida: Plasmodiidae) can produce terpenes [93] thanks to the metabolic pathways occurring in their apicoplast, a plastid organelle with evolutionary origins similar to the chloroplasts in plants [171]. These *Plamodium*-derived terpenes have been suggested to be semiochemicals involved in mediating the preference for mammalian hosts in anopheline mosquitoes [93].

The CH profile is altered due to *Plasmodium* infection in the malaria vector *Anopheles albimanus* Wiedemann, 1820 (Diptera: Culicidae), leading to a reduction in the total number of CHs [94]. Considering that CHs have been widely recognized for their multifaceted roles in insect chemical communication and their impact on reproductive biology [140,172,173,174,175,176,177,178,179], which includes their hypothesized role as attractiveness signals for mating in other mosquito species [180], Claudio-Piedras and colleagues suggested that *Plasmodium* infection could potentially influence the physiology and behavior in *An. albimanus* [94].

Beyond the biological relevance of these findings, there is also an important practical application, as the CH profile can be exploited as a marker of *Plasmodium* infection. This is particularly feasible, given that gas chromatography (GC) coupled with mass spectrometry (MS) analysis to trace the CH profile does not require sample preservation, and the hexane-based CH extraction is non-destructive, allowing an afterwards use of the mosquitoes [94].

### 5.2. Phylum Euglenozoa

Tsetse flies (*Glossina* genus) are vectors of African trypanosomes causing sleeping sickness in humans and nagana in livestock, with devastating public health and economic effects in Africa [181,182]. Reproductive biology in these species has been widely investigated [55,183,184,185,186,187,188,189,190,191,192,193,194,195,196,197,198,199], not only to develop novel tsetse control approaches but also for its unique features related to adenotrophic viviparity (i.e. maternal nourishment of the progeny in the uterus followed by live birth of the larva) [200]. However, only very recently, volatile chemicals were shown to affect mating behavior in tsetse flies [95]. In particular, the three chemicals methyl palmitoleate (MPO), methyl oleate (MO), and methyl palmitate (MP) were found to be produced by females of *Glossina morsitans morsitans* (Diptera: Glossinidae), with MPO eliciting a particularly strong behavioral response (i.e., attraction) in males. Thus, the authors suggested that MPO may play the role of a volatile sex attractant in *G. m. morsitans*, acting as an aphrodisiac to elicit sex-specific behavior effects. Interestingly, in the same study, the authors found that trypanosome infection affected the chemical profile of mated individuals of both sexes. In particular, 21 volatile compounds were present in extracts of the body wash of infected flies but absent from the uninfected controls. As mentioned above, malaria parasites were previously shown to produce chemicals in infected mosquitoes [93]. One of these compounds, α-pinene, is common between *Plasmodium*-infected mosquitoes and trypanosome-infected tsetse flies. In addition, the *G. m. morsitans* antennal odorant receptor GmmOr35 was previously shown to be responsive to α-pinene [201], suggesting that other flies may detect trypanosome infection, with potential effects on mating behavior. Notably, in infected tsetse females, the fecundity is reduced [96], similar to what occurs in *Plasmodium*-infected mosquitoes, which produce fewer eggs than their uninfected counterparts [202,203]. The intriguing question of whether the fly or the parasite produces these chemicals remains unanswered.

### 5.3. Phylum Platyhelminthes

Early studies described that, in the intermediate coleopteran host *Tenebrio molitor* Linnaeus, 1758 (Coleoptera: Tenebrionidae), the infection with metacestodes of the rat tapeworm, *Hymenolepis diminuta* (Cyclophyllidea: Hymenolepididae), impairs vitellogenesis and reduces host fecundity through the modulation of juvenile hormone-regulated events [204,205,206]. Subsequently, Hurd and coworkers further described this infection model, and, for the first time, reported that a parasite can affect host sex pheromone production [97]. Indeed, the research team showed that parasitization strongly decreases the non-volatile copulatory release of pheromones in *T. molitor* females and negatively affects the male response to pheromones [97]. These effects may contribute to the observed delay in oviposition and fecundity reduction associated with the metacestodes–coleoptera relationship.

Among the intermediate insect hosts exploited by *H. diminuta* is also the red flour beetle, *Tribolium castaneum* (Herbst, 1797) (Coleoptera: Tenebrionidae). In this case, infection with the rat tapeworm leads to altered behaviors in the beetles [98], diminished male sperm precedence [99], and the production of defensive compounds, although among-strain variations have been reported [100]. Intriguingly, it has been demonstrated that *H. diminuta* infection significantly reduces *T. castaneum* male fitness [101]. Importantly, this reduction is not attributable to female mate choice against infected mates, opening the way to novel investigations aimed at addressing unanswered questions concerning the mechanisms underlying this phenomenon. Infection of *T. confusum* Jacquelin du Val, 1868 (Coleoptera: Tenebrionidae) by *H. diminuta* was shown to lead to reduced survival, fecundity, and mating vigor, as well as carbohydrate metabolism and pheromone response [102,103,104]. More recently, an upregulation of pheromone binding proteins was also reported [207], similar to the effect described for virus-infected *D. melanogaster* [75]. Whether such an upregulation increases the predation susceptibility of infected hosts (thus favoring parasite persistence) or confers protection to the host is still an open question.

### 5.4. Phylum Nematoda

Infection by the entomopathogenic nematode *Heterorhabditis bacteriophora* (Poinar, 1975) (Strongylida: Heterorhabditidae) was shown to change the behavior of healthy larvae of *Diabrotica virgifera* (Coleoptera: Chrysomelidae), the Western corn rootworm [105]. Nematode-infected *D. virgifera* cadavers attract uninfected rootworm larvae, increasing the infection rate and the nematode’s reproductive success. This is achieved by emission from the nematode-infected rootworms of volatile chemicals such as butylated hydroxytoluene (BHT), a compound that is not common in nature and functions as an attractant to healthy insects. Indeed, it is important to note that approaching a nematode-infected cadaver poses risks for the rootworm. It is conceivable that BHT triggers a response in the rootworm by mimicking the activity of chemicals involved in host selection [105]. In addition, the volatiles released from insect cadavers infected by entomopathogenic nematodes can increase the resistance of plants to insect herbivores [208]. Interestingly, these are examples of a double ability of volatiles to affect the behavior of insects directly or indirectly by modifying plant responses. At present, however, the molecular mechanism through which BHT is produced in the rootworm cadavers and the nematode-specific factors triggering this process remain to be explored. Further investigation in this regard is warranted, as it carries significant practical implications for pest control and raises interesting evolutionary and ecological questions about the adaptive nature of this insect behavior.

## 6. Conclusions

**Figure 1:**
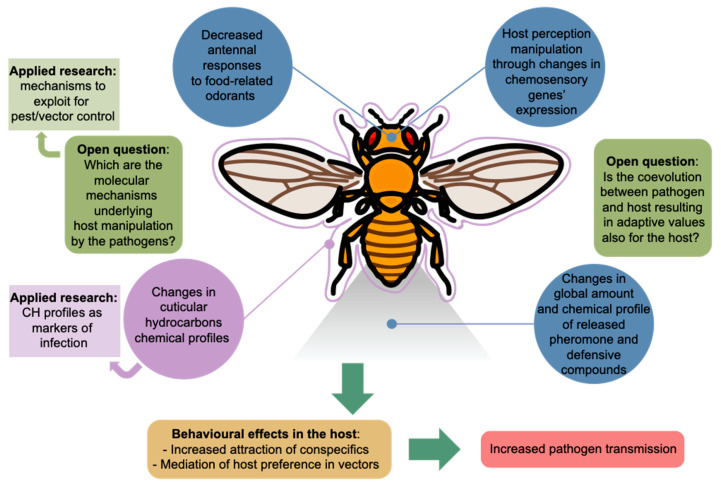
Reported effects of pathogen infection on insect chemical communication. The diagrammatic representation of a *D. melanogaster* female was adapted from the fly_female_adult_from_a_overhead_view icon by DBCLS https://togotv.dbcls.jp/en/pics.html, accessed on 27 September 2023, licensed under CC-BY 4.0 Unported https://creativecommons.org/licenses/by/4.0/.

Research on the roles that pathogens play in insect–host chemical communication is still in its early stages. Thus far, only a limited number of effects have been described, as summarized in Figure 1.

Most studies, especially those focused on viral and fungal infections, have investigated these phenomena in light of the evolutionary advantages for the pathogens. A crucial challenge is to put these studies in the frame of dynamic and reciprocal relationships that shape the co-evolution between pathogen and host and may result in adaptive values for the host, as shown in other animal models [38]. In certain cases, pathogen infection leads to restrain reproduction to sustain antipathogen immune response and, as a consequence, subsequent reproductive opportunities [209]. This scenario is more likely to unfold when there is a high infection pressure; whereas, in the presence of a low pathogen prevalence, natural selection may lean towards increased allocation to reproduction [210]. Insect hosts are able to compromise key life history traits (e.g., growth, homeostasis, reproduction) to fight pathogen infection. In parallel, pathogens display trade-offs between virulence and transmission, or survival outside the insect host and transmission (see [211] for a review). This underscores the necessity of expanding our knowledge on the eco-evolutionary dynamics of host–pathogen systems.

In most cases, the genetic bases and the molecular (especially neuronal) mechanisms underlying host manipulation by the pathogens are still completely unknown. In this context, a subject that may deserve special attention is the impact of pathogen infection on pheromone production, which, in turn, is known to influence hormonal responses, at least in certain species. For instance, in social insects such as honey bees, queens can shape worker behavior and colony dynamics through queen pheromones [212,213,214,215,216,217,218,219,220,221,222]. In the case of *Apis mellifera*, exposure to the QMP has been shown to modulate ecdysteroid titers in workers [223]. These hormones can influence behavior and physiology by regulating gene expression in the brain through interaction with ecdysteroid receptors [224,225]. Considering the established roles of ecdysteroid hormones in immunity regulation, particularly in antipathogen defense [226,227,228], studies aimed at unraveling the dynamic interplay among pheromone, hormonal response, and pathogen infection would be particularly intriguing.

As previously mentioned, a wider understanding of the mechanisms underlying pathogen-mediated behavioral changes will also be beneficial for an applied perspective. Indeed, a deeper knowledge of the interactions between pathogen and host at the molecular level will reveal novel targets to be exploited to manipulate the behavior of insect agricultural pests and disease vectors, thus expanding the insect control toolbox in the field.

Finally, the influence of pathogens on the chemical communication of herbivorous insects should also be examined within the broader context of other trophic levels, such as their interaction with host plants. Plants play a role in modulating interactions between insect herbivores and their pathogens, for instance, by employing insect pathogens as defensive tools against herbivory (refer to [229,230] for reviews on this topic). Investigating how insect pathogens integrate into a multitrophic framework provides an interesting perspective for the study of insect–pathogen relationships.

## Figures and Tables

**Table 1:** Insect pathogens and their impact on the host chemical communication at the genomic, physiological, and behavioral levels.

Pathogens (Superkingdom/Kingdom)	Insect Host Species (Order)	Effects on Host	References
**Bacteria**			
*Pseudomonas entomophila* *Serratia marcescens* *Pectobacterium carotovorum*	*Drosophila* spp. *Aedes aegypti* (Diptera)	Increased pheromone emission	[70]
**Viruses**			
Hz-2V	*Helicoverpa zea* (Lepidoptera)	Females: calling behavior, increased pheromone production, stronger male attraction. Males: cannot produce pheromonostatic peptide	[8,71,72]
SeMNPV ^1^	*Spodoptera exigua* (Lepidoptera)	Changes in expression of larval odorant receptors resulting in behavioral responses to its ligands	[73]
AcMNPV ^2^	*Spodoptera frugiperda* *Trichoplusia ni* (Lepidoptera)	Co-induction of *Desaturase1* and *bond* (pheromone production/perception and conspecific signaling). Enrichment in functions related to fatty acid biosynthesis and pheromone metabolisms	[74]
DCV ^3^	*Drosophila* spp. (Diptera)	Induction of pherokine-2	[75]
DENV-2	*Aedes aegypti* (Diptera)	Effects on the expression of chemosensory-related genes involved in the regulation of blood feeding	[76]
RVFV ^4^	*D. melanogaster* (Diptera)	Changes in odor response and activity	[77]
RhPV ^5^	*Rhopalosiphum padi* (Hemiptera)	Increased sensitiveness to the alarm pheromone	[78]
**Fungi**			
*Beauveria bassiana*	*Triatoma infestans* (Hemiptera)	Differences in the profile of volatile organic compounds. Increased expression of genes involved in the synthesis of volatile short-chain fatty acids	[79,80]
*Beauveria bassiana* *Beauveria brongniartii*	*Melolontha melolontha* (Coleoptera) *Ostrinia nubilalis* (Lepidoptera)	Alterations in cuticular hydrocarbon profile	[81]
*Nosema* spp.	*Apis mellifera* (Hymenoptera)	Workers: Increased production of the primer foraging pheromone ethyl oleate. Increased flight activity and mortality. Queen: Increased vitellogenin titer, antioxidant capacity, and mandibular pheromones. Increased synthesis of alarm pheromone component. Alterations in cuticular hydrocarbon profile	[82,83,84,85,86]
*Paranosema (Nosema) locustae*	*Locusta migratoria manilensis* (Orthoptera)	Inhibition of aggregation behavior due to decreased production of aggregation pheromone	[87,88]
*Pandora neoaphidis*	*Acyrthosiphon pisum* (Hemiptera)	Increased release of alarm pheromone	[89]
*Conidiobolus coronatus*	*Blatta orientalis* (Blattodea)	Increased production of cuticular hydrocarbons and other surface compounds	[90]
*Metarhizium brunneum*	*Lasius neglectus* (Hymenoptera)	Changes in cuticular hydrocarbon profile and alteration in volatile chemical cues emission	[91]
*Entomophthora muscae*	*Musca domestica* (Diptera)	Production of a mixture of volatile sesquiterpenes altering the CH profile in female cadavers	[92]
**Eukaryota**			
***Phylum Apicomplexa***			
*Plasmodium falciparum*	*Anopheles gambiae* (Diptera)	Production of terpenes putatively mediating mammalian host preference	[93]
*Plasmodium berghei*	*Anopheles albimanus* (Diptera)	Altered cuticular hydrocarbon profile	[94]
***Phylum Euglenozoa***			
*Trypanosoma brucei*	*Glossina morsitans morsitans* (Diptera)	Altered chemical profiles of infected mated individuals. Reduced fecundity	[95,96]
***Phylum Platyhelminthes***			
*Hymenolepis diminuta*	*Tenebrio molitor* (Coleoptera)	Decreased production of non-volatile copulatory pheromone in females. Negative effects on male response to pheromone	[97]
	*Tribolium castaneum* (Coleoptera)	Altered behavior. Decreased male sperm precedence and fitness. Decreased production of defensive compounds	[98,99,100]
	*Tribolium confusum* (Coleoptera)	Reduced survival, fecundity, mating vigour, carbohydrate metabolism, and pheromone response. Upregulation of pheromone binding proteins	[101,102,103,104]
***Phylum Nematoda***			
*Heterorhabditis bacteriophora*	*Diabrotica virgifera* (Coleoptera)	Increased emission of volatiles such as butylated hydroxytoluene	[105]

^1^ *Spodoptera exigua* multiple nucleopolyhedrovirus. ^2^ *Autographa californica* multiple nucleopolyhedrovirus. ^3^ *Drosophila* C virus. ^4^ Rift Valley fever virus. ^5^ *Rhopalosiphum padi* virus.

## Data Availability

Not applicable.
